# Identification of Black Bean Geographical Origins Using Portable NIR Spectroscopy Combined with Fuzzy Orthogonal Linear Discriminant Analysis and Its Kernel Extension

**DOI:** 10.3390/foods15203670

**Published:** 2026-10-15

**Authors:** Jiajun Dong, Xiaohong Wu, Chenao Xie, Jing Zhou, Bin Wu

**Affiliations:** 1 School of Electrical and Information Engineering, Jiangsu University, Zhenjiang 212013, China; 2222407079@stmail.ujs.edu.cn (J.D.); 2222207011@stmail.ujs.edu.cn (C.X.); zj1574437386@163.com (J.Z.); 2 High-Tech Key Laboratory of Agricultural Equipment and Intelligence of Jiangsu Province, Jiangsu University, Zhenjiang 212013, China; 3 Department of Information Engineering, Chuzhou Polytechnic, Chuzhou 239000, China; 4 School of Computer Science and Engineering, Southeast University, Nanjing 211189, China

**Keywords:** black beans, near-infrared spectroscopy, non-intrusive detection, K-nearest neighbor, kernel function

## Abstract

Since different geographical origins of black beans have different nutritional and economic values, their classification is of great significance in the field of agriculture. Therefore, this study aims to develop a classification model for black bean origin identification based on chemometric analysis of near-infrared (NIR) spectral data. Due to the overlap and complex distribution characteristics of raw NIR spectral data, traditional linear discriminant analysis algorithms are unable to extract effective discriminative information from them. To overcome these limitations and improve the performance of the algorithms, fuzzy set theory was incorporated into orthogonal linear discriminant analysis (OLDA), and the fuzzy OLDA (FOLDA) was proposed to extract discriminant features from the NIR absorption spectra of black beans. FOLDA enhances sample diversity and better handles noisy data. Furthermore, by combining FOLDA with a linear kernel function, the kernel FOLDA (KFOLDA) algorithm was proposed to address the challenge of linear algorithms in processing nonlinear data. KFOLDA not only effectively captures and processes nonlinear information within the data but also significantly improves the model’s classification accuracy. The experimental results show that the K-nearest neighbor (KNN) classifier achieved classification accuracies of 86.4% and 95.5% for FOLDA and KFOLDA, respectively. Thus, the proposed model demonstrates satisfactory classification accuracy and performance metrics for distinguishing different geographical origins of black beans.

## 1. Introduction

Black beans, as a medicinal and healthy legume food, are popular among domestic and international consumers because of their richness in protein, amino acids, anthocyanins and other nutritional elements [1]. Black beans are produced in many parts of China, with the main production areas concentrated in Dongbei, Xinjiang, Shanxi and Sichuan. In recent years, due to the improvement of individual living standards, the requirements for nutritional ingredients in food have also increased. Black beans from different geographical origins have different nutrients [2]. Currently, the market is flooded with various types of black beans that have similar appearances, leading to the emergence of fraudulent practices. Merchants confuse high-quality black beans with low-quality black beans, making it difficult to avoid adulterated sales in the black bean market, which damages the interests of consumers. Furthermore, since the nutritional composition of black beans depends on their origin, it is feasible to develop related products and extract functional ingredients by leveraging their distinct properties [3]. Such an approach would be advantageous for processing departments as it would help in improving product utilization and enhancing the extraction purity of relevant nutritional components. Hence, it is imperative to establish a fast, non-intrusive, and accurate classification model for identifying different geographical origins of black beans.

Human sensory and chemical methods are the traditional methods for identifying black bean origins, and chemometrics-based data analysis has been further applied to assist origin discrimination [4,5]. Utilizing the sense organs of the human body to distinguish black bean species will waste a lot of labor and material resources, and the identification cycle is long and inefficient [6,7,8]. In recent years, some researchers have also used chemical methods to identify the varieties of related beans [9,10]. While the chemical method can obtain high classification accuracy, the cost of conducting experiments is prohibitively expensive and not practical for identifying large quantities of samples. With the rise in non-intrusive detection, near-infrared (NIR) spectroscopy technique is also extensively employed in food, agricultural products and other fields [11,12,13]. Sun et al. proposed a system for rapid differentiation of black bean varieties based on NIR hyperspectral images [14]. Portable NIR spectrometers are flexible, affordable, and easy to use, making them useful in agriculture, food quality assessment, and pharmaceutical analysis. A portable micro-NIR spectrometer was used in combination with multivariate calibration of partial least squares (PLS) and PCA to detect adulteration in Arabica coffee beans, which helped in controlling the quality of the beans [15].

Fuzzy set theory is a method that uses fuzzy mathematics to process and identify observations. Fuzzy recognition is capable of precisely characterizing the variety of information present in samples, reducing the impact of overlapping samples on classification results, and enhancing the classification ability of the model [16,17]. As a result, fuzzy classification has been widely used in many fields. A study incorporated fuzzy set theory into the improved linear discriminant analysis (iLDA) and proposed a fuzzy iLDA algorithm, which improved the classification model’s accuracy to 94.4% [18]. Abe proposed a fuzzy support vector machine (FSVM) for multi-label classification, which further optimized the classification model [19]. Few previous studies have applied fuzzy set theory in combination with feature extraction algorithms for the classification of black bean origins. Using linear discriminant analysis (LDA) to extract features faces the small sample size problem, which leads to more redundant information in the transformation matrix. Orthogonal linear discriminant analysis (OLDA) leverages the orthogonal relation between feature vectors to mitigate the risk of a non-invertible covariance matrix, thereby effectively addressing the small sample size (SSS) problem associated with LDA [20]. By incorporating fuzzy set theory into OLDA, fuzzy orthogonal linear discriminant analysis (FOLDA) can handle the SSS problem and provide a more nuanced understanding of the data. The fuzzy membership function enables FOLDA to assign degrees of membership to each sample, instead of solely classifying them as belonging to a particular group. This allows for a more accurate representation of the diversity within the data. FOLDA is an innovative fuzzy type of OLDA that is designed to acquire valid classification information from data containing overlapping and noisy data.

The theory and algorithms of machine learning and statistics can handle linear problems well, but many practical problems in data classification require the use of non-linear methods for classification or prediction [21]. The kernel function serves the purpose of mapping the original samples in feature space from the low dimension to the high dimension, where the samples can be more easily classified. The kernel method has decent performance in extracting nonlinear feature information from data. For example, Al-Dabagh et al. utilized kernel discriminant analysis (KDA) to perform feature extraction from input images and proposed a face recognition system using SVM and KNN [22]. Finally, their system achieved a high recognition rate. Radial basis function (RBF) was introduced into extreme learning machine (ELM) and combined with 2D PCA to classify hyperspectral images [23]. This kernel-based model significantly improved the classification performance. This study combines kernel functions with FOLDA, which can solve the problem that linear algorithms have difficulty classifying non-linear data. The kernel-based model can further perform feature extraction from the NIR spectra of black beans, thus improving the identification accuracy.

This study proposed the FOLDA algorithm to mitigate the impact of noisy data on classification models and showcased the diversity of sample classification. Furthermore, the KFOLDA algorithm was introduced by integrating a kernel function to overcome the limitation of linear algorithms in handling nonlinear data. This study aims to introduce kernel functions and fuzzy theory into the OLDA algorithm to present FOLDA and KFOLDA for the identification of black bean origins. Initially, a portable NIR spectrometer was utilized to acquire NIR spectra from five different origins of black bean samples. Preprocessing algorithms were subsequently applied to eliminate noise and scattering information from the collected data. The dimensionality of the black bean data was decreased by PCA. Subsequently, LDA, OLDA, FOLDA and KFOLDA were respectively utilized in extracting discriminatory information from the spectral data of different origins of black beans. Finally, the variety of black bean samples was determined by the KNN algorithm, and the final classification accuracy was calculated from the KNN results.

## 2. Materials and Methods

### 2.1. Sample Preparation

**Figure 1:**
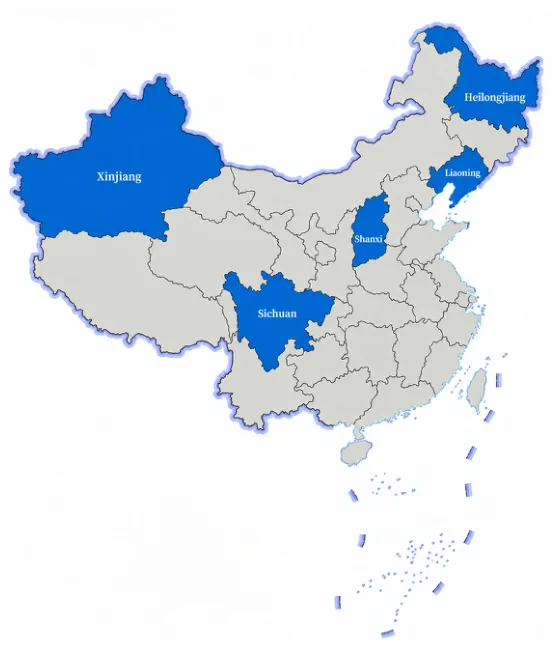
Geographical locations of sample origins for black beans.

The geographical locations of black bean sample origins are shown in Figure 1. All samples from five different geographical origins, including Liaoning, Heilongjiang, Xinjiang, Sichuan, and Shanxi, were prepared for the experiment. These five producing regions differ substantially in geography, climate and soil conditions. Heilongjiang and Liaoning feature a temperate monsoon climate with long cold winters, sufficient sunshine hours during the growing season, and fertile black chernozem soil rich in organic matter. Shanxi belongs to the temperate continental monsoon climate, with large diurnal temperature variation and mainly loess-derived soils. Xinjiang is characterized by an arid continental climate with low precipitation, strong solar radiation, large day-night temperature differences, and desert-grey-brown soils. Sichuan has a subtropical monsoon climate with abundant rainfall, high humidity, and mostly purple soils. Such regional disparities in temperature, solar radiation, precipitation, soil type, soil organic matter and mineral element availability can modulate protein, anthocyanin and carbohydrate synthesis in black beans, ultimately generating differences in chemical composition and nutritional quality among black beans from different geographical origins.

**Figure 2:**
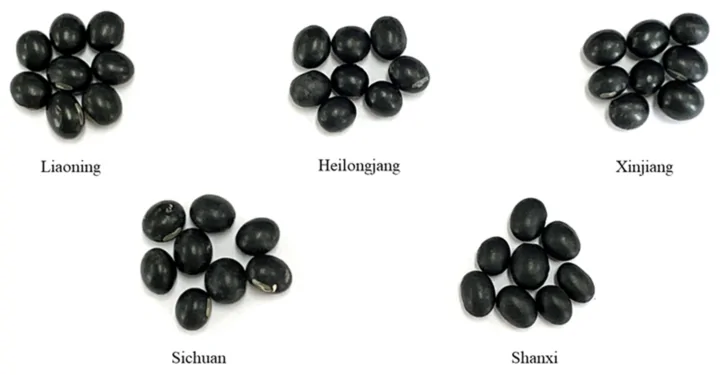
Black bean samples from five different origins.

Five origins of black bean samples are shown in Figure 2. These samples were all purchased from the online shopping platform, Taobao, and were directly sourced from their places of origin. The purchased black bean samples should be stored in a sealed container at normal temperature to prevent problems such as oxidation and deterioration. In this experiment, the number of black bean samples used for each variety is 80, and the total number of samples for all varieties amounts to 400. These samples were divided into training and test sets in a ratio. In this study, 58 samples from each class were chosen for training, and the rest for testing. It is necessary to ensure that the surface of the selected sample is smooth and intact, which is more conducive to measurement. At the same time, through measurement and weighing operations, the experimenters ensured that each black bean sample satisfied the following conditions: a length of 5–6 mm, a weight of 0.5–0.7 g, and a smooth surface and standard shape.

### 2.2. Spectra Collection

**Figure 3:**
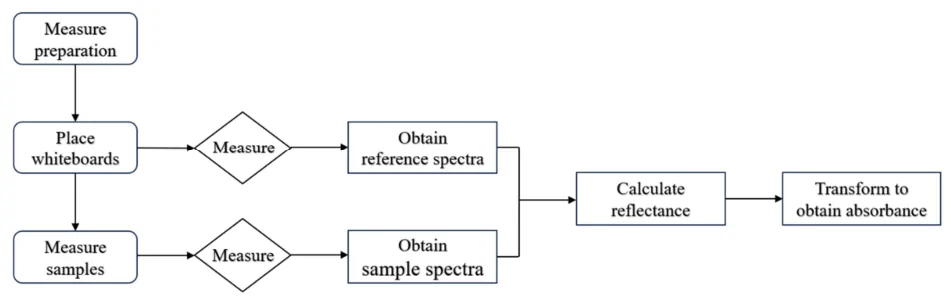
The measurement principle of NIR spectrometer.

**Figure 4:**
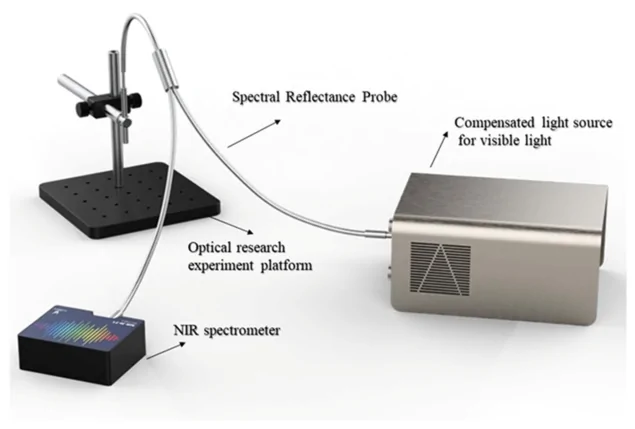
The combination of instruments used in the experiment.

The NIR-M-F1-C portable NIR spectrometer (made by Shenzhen Puyan Internet Technology Co., Ltd., Shenzhen, China) was used to acquire NIR spectra of black bean samples from five different regions. The purchased black beans were stored in sealed containers under refrigeration at 4°C to avoid oxidation and deterioration. The sample testing cycle was short. Such short-term storage conditions could inhibit oxidation and deterioration, and their interference with NIR spectral signals was considered negligible. Its measurement principle is shown in Figure 3. The portable NIR spectrometer offers significant advantages, such as being lightweight, low-cost, and compact in size, as illustrated in Figure 4. Additionally, the operation process is simple, and it can be used for routine analysis by plant breeders and growers [24]. For the spectrometer, the wavelength ranges from 900 nm to 1700 nm. Throughout the experiment, the humidity was maintained at approximately 25–30% and the temperature was kept at 25 °C. Before collecting the spectra, the spectrometer needed to be warmed up for half an hour in advance to avoid large fluctuations in the measurement. The collected data were saved using the software DLP NIRscan Nano GUI v2.1.0. The result of one scan was obtained by averaging the values from 6 exposures, with each acquisition taking approximately 3.64 s. Each sample was scanned three times, and the mean of these scans was taken as the final data for the sample. Following data collection, the resulting dataset had a final dimensionality of 400.

### 2.3. NIR Spectra Preprocessing

**Figure 5:**
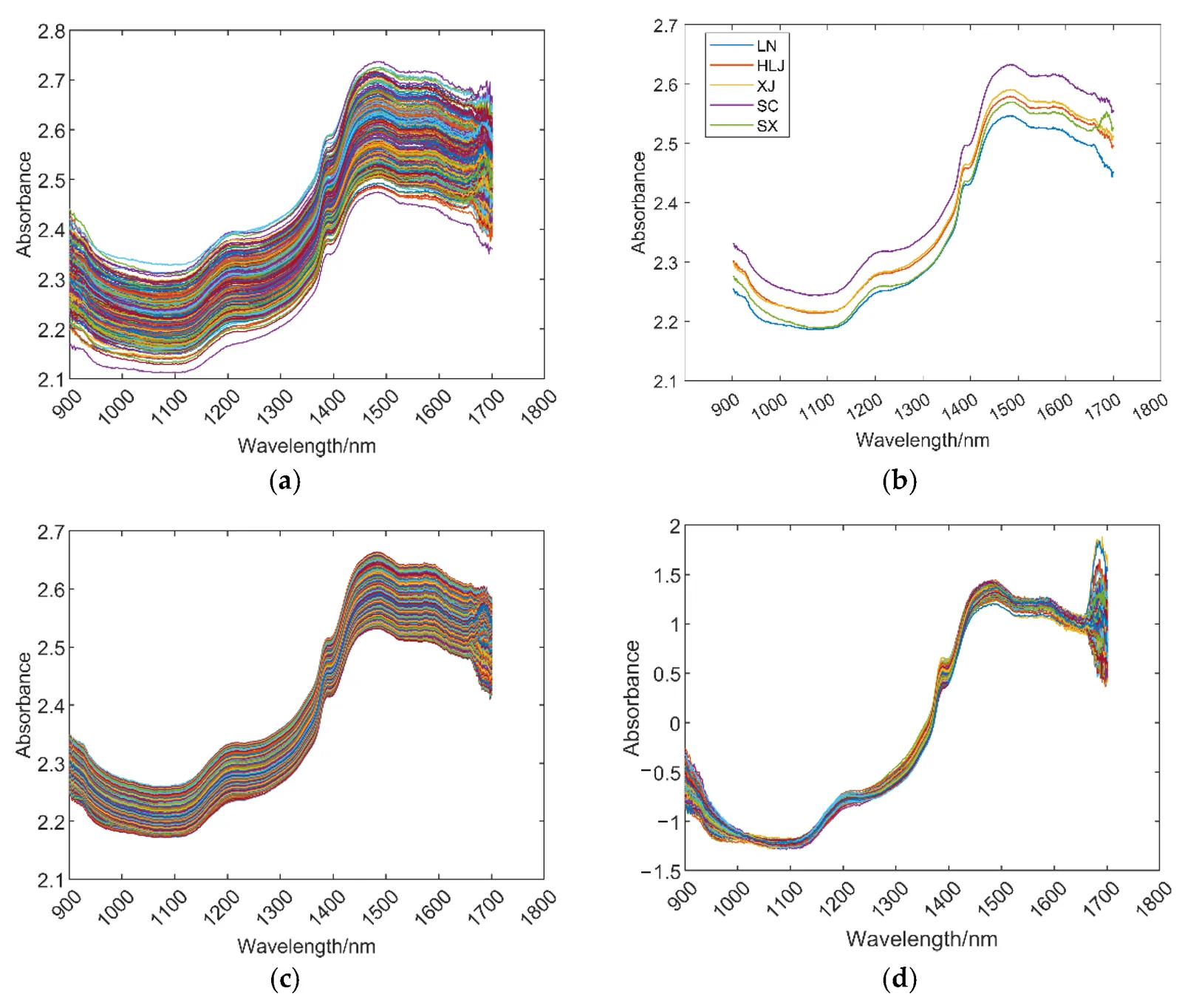

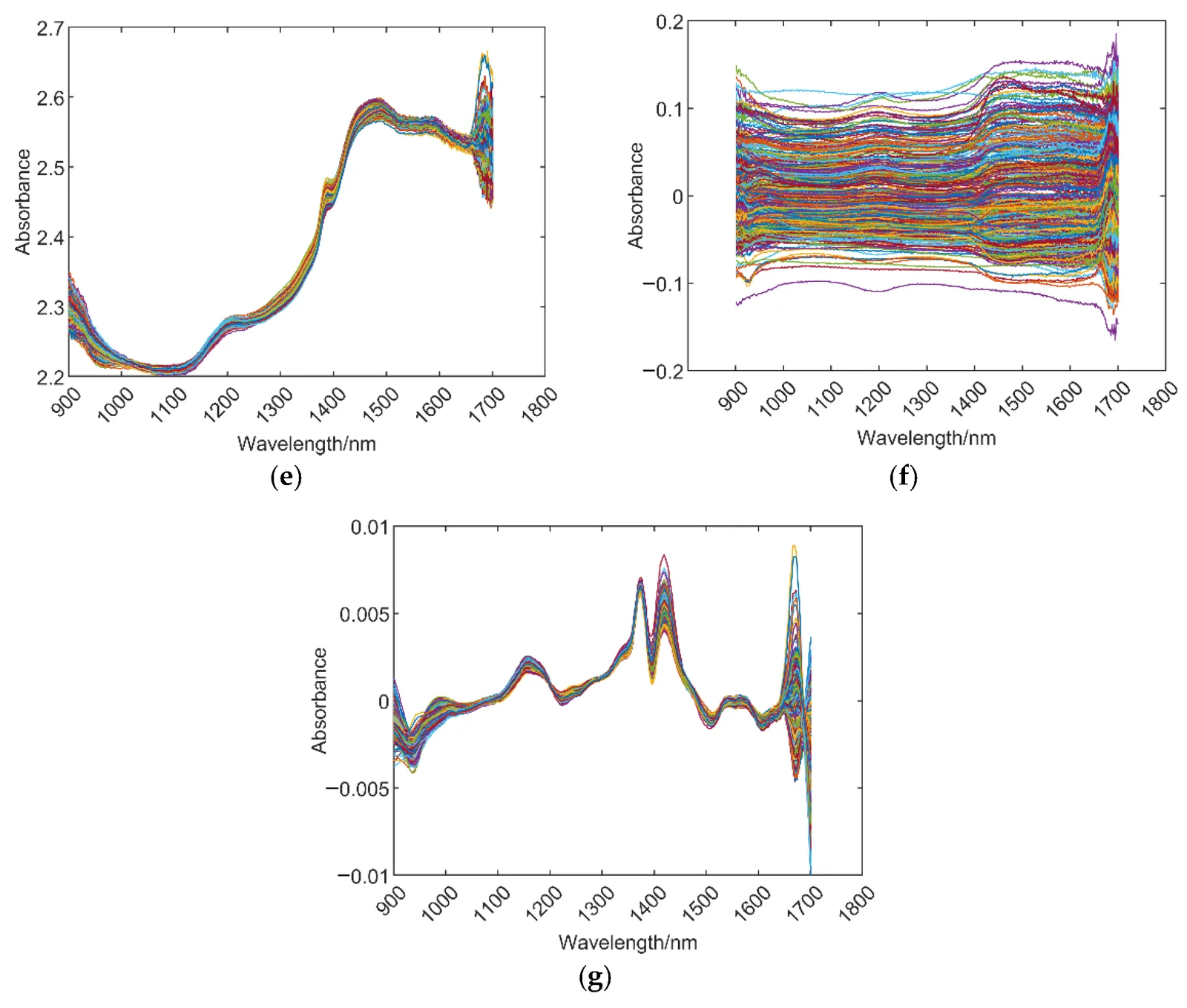
The original and preprocessed NIR spectra of black bean samples: (**a**) The original NIR spectra. (**b**) The average NIR spectra of each origin of black beans. (**c**) SG, (**d**) SNV, (**e**) MSC, (**f**) MC, and (**g**) 1st D.

The original NIR spectra of the black bean sample data are shown in Figure 5a. In addition to the sample characteristics required by the experiment, the original data were also blended with some noise and scattering information [25]. Thus, preprocessing of the raw spectral data was deemed necessary to enhance the model’s performance. In this experiment, five preprocessing algorithms, Savitzky–Golay (SG), standard normal variation (SNV), multiple scattering correction (MSC), mean centering (MC) and first-order derivative (1st D), were respectively employed to process the raw NIR spectra. The experiment then compared the results of these algorithms to determine which one produced the most accurate classification model. When using the SG filter, we need to follow its syntax rule y = sgolayfilt (x, order, framelen), noting the two parameters: polynomial order and box length framelen. The filter’s order must be less than the frame length, which is required to be an odd number. If the order equals framelen − 1, the filter will not produce a smoothing effect. In this experiment, the order was 1, and the frame length was 19. Different preprocessing algorithms have different functions and capabilities. SG is mainly used for the reduction in noise interference in data and keeps the spectral shape and width unchanged [26]. MSC can effectively remove the spectral difference arising from variations in scattering levels, thereby strengthening the relationship between spectra and data [27]. SNV is employed to reduce the impact of uneven sample particle size and non-specific scattering on particle surfaces [28]. The spectral graphs of black bean data after being preprocessed by the five preprocessing methods are shown in Figure 5c–f.

### 2.4. Chemometrics Methods

#### 2.4.1. Principal Component Analysis

After preprocessing, the NIR spectra of the black bean samples were still 400-dimensional data. Raw NIR spectra often include redundant variables and noise, which increase computation and can reduce classification accuracy. In this study, PCA was applied to reduce the dimensionality of the spectral data. PCA can convert the high-dimensional data to low-dimensional data to find an optimal transformation matrix. With maximum variance preserved in the low-dimensional representation, fewer eigenvalues were used to retain most of the original information, thereby enhancing model accuracy and boosting efficiency.

#### 2.4.2. Linear Discriminant Analysis

Discriminant feature extraction is a crucial procedure for the whole study. On the one hand, it can carry out the separation of signal and noise; on the other hand, it is mainly devoted to obtaining discriminant feature information from the data [29]. The Fisher criterion function was first proposed by Fisher, a famous British statistician, in 1936. LDA finds the optimal transformation vectors that satisfy the criteria of minimizing intra-class distance and maximizing inter-class distance in the low-dimensional feature space. LDA aims to reduce dimensionality and extract features from NIR data [30].

#### 2.4.3. Foley–Sammon Transformation

Foley–Sammon transformation (FST) is a classic method for feature extraction, which was proposed by Foley and Sammon [31]. FST extends Fisher’s linear discriminant analysis by requiring the extracted discriminant vectors to be orthogonal while optimizing the Fisher criterion. Compared with LDA, FST can extract additional orthogonal discriminant vectors.

#### 2.4.4. Uncorrelated Discriminant Transformation

The discriminant vectors obtained by FST are not necessarily uncorrelated. Uncorrelated discriminant transformation (UDT) addresses this limitation by imposing an uncorrelated constraint [32]. Compared to FST, UDT can extract more valuable feature information. Moreover, UDT can significantly enhance the performance during feature extraction when facing the SSS problem. Through a novel approach to calculating the discriminant vector, UDT can avoid the problem of feature extraction failure caused by a singular within-class scatter matrix, while maintaining the irrelevance of the discriminant vectors.

#### 2.4.5. Orthogonal Linear Discriminant Analysis

Several improved LDA algorithms have been proposed to address the SSS problem. Among them, Ye and Yu proposed a new discriminant analysis algorithm, namely OLDA, which used the pseudo-inverse matrix to substitute the inverse matrix in the Fisher discriminant criterion to deal with the SSS problem [20]. The main characteristic of OLDA is that the discriminant vectors obtained satisfy the orthogonality principle. Furthermore, OLDA is particularly adept at handling scenarios where the scatter matrices involved are not invertible, effectively circumventing the issue of matrix singularity.

#### 2.4.6. The Proposed Fuzzy Orthogonal Linear Discriminant Analysis

Based on the fuzzy concept, our proposed fuzzy feature extraction method, called FOLDA, not only overcomes the SSS problem of the traditional LDA algorithm but also explains the diversity of sample categories. Considering the fuzzy membership function, we can take into account the correlation and differentiation between different features, thus providing a more comprehensive description of the relationship between features and extracting more effective discriminative information. The FOLDA feature extraction algorithm is described in detail below.

Given sample matrix $X\in R^{l\times n}$, l denotes the feature dimension and n is the total number of samples. Let c be the number of classes. According to fuzzy membership $u_{ij}$, three fuzzy weighted scatter matrices are constructed: fuzzy total-scatter matrix $H_{ft}$, fuzzy between-class scatter matrix $H_{fb}$, and fuzzy within-class scatter matrix $H_{fw}$. These three matrices are defined in a related study [29].

FOLDA aims to seek a column-orthogonal transformation matrix Q, such that the projected features achieve large fuzzy between-class dispersion while suppressing fuzzy within-class dispersion. The numerical solution procedure of FOLDA is given as follows:

Step 1. Perform singular value decomposition on the fuzzy total-scatter matrix:

(1) $$H_{ft}=G\Sigma O^{T}$$

where $G\in R^{l\times l}$ and $O\in R^{n\times n}$ are orthogonal matrices, and $\Sigma \in R^{l\times n}$ is the singular-value diagonal matrix. Denote $t=rank(H_{ft})$. Take the first t columns of G as $G_{1}$, and extract the invertible t×t top-left sub-matrix of Σ denoted as $\Sigma_{t}$.

Step 2. Construct intermediate matrix B:

(2) $$B\;=\;\Sigma_{t}^{-1}G_{1}^{T}H_{fb}$$

Carry out singular-value decomposition for B:

(3) $$B\;=\;P\Lambda O^{T}$$

P is the left-singular-vector matrix, A is the singular-value diagonal matrix, and $O^{T}$ is the right-singular-vector matrix.

Step 3. Compute intermediate matrix Y:

(4) $$Y\;=\;G_{1}\Sigma_{t}^{-1}P$$

Extract the first q columns of Y to obtain $Y_{q}\in R^{l\times q}$, where $q=rank(H_{fb})$.

Step 4. Apply QR decomposition for $Y_{q}$:

(5) $$Y_{q}=QR$$

$Q\in R^{l\times q}$ is the column-orthogonal matrix and R is upper-triangular. The matrix Q serves as the feature transformation matrix of FOLDA.

#### 2.4.7. Kernel Fuzzy Orthogonal Linear Discriminant Analysis

**Figure 6:**
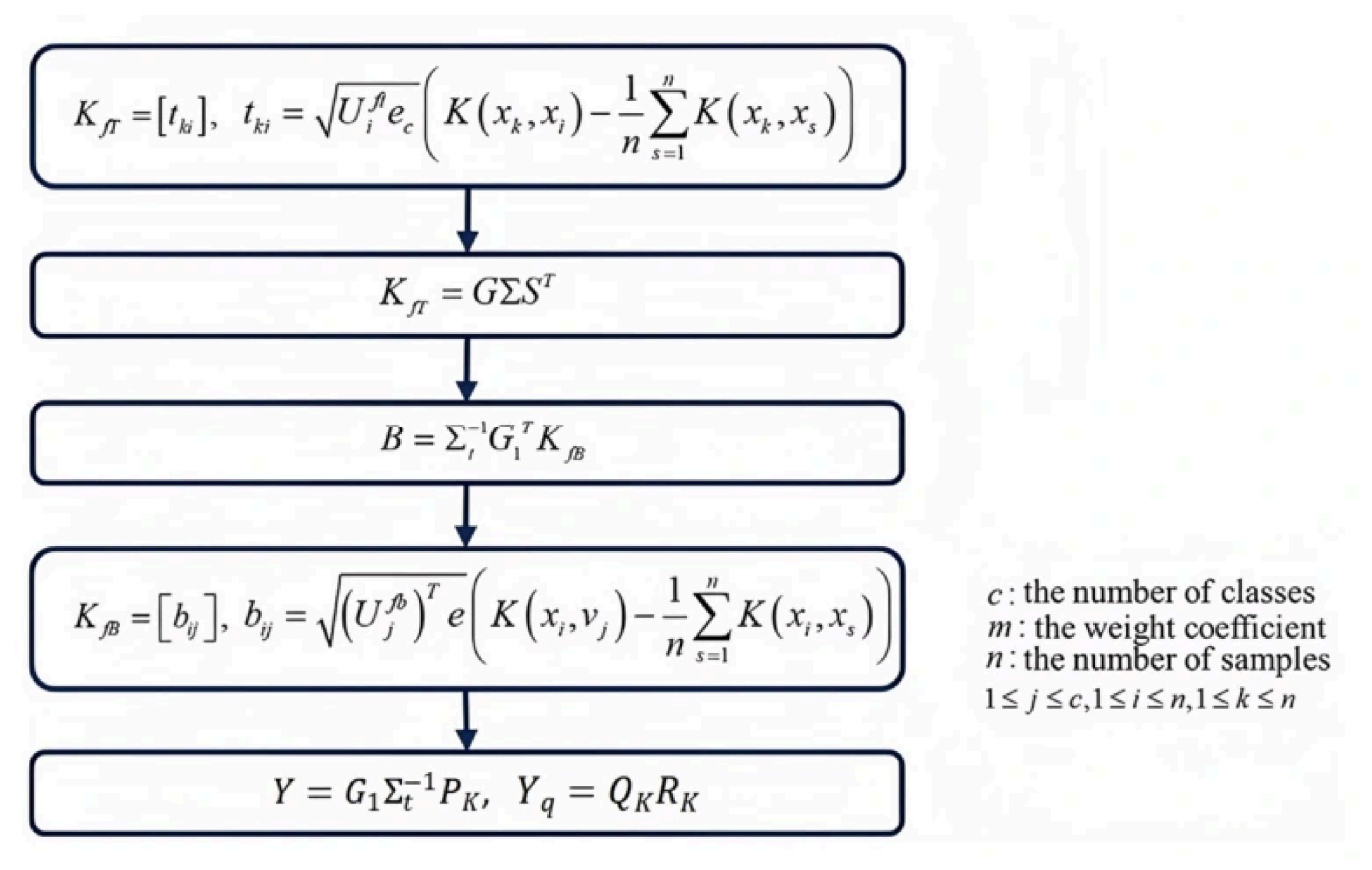
The derivation process of KFOLDA.

FOLDA is more suitable for extracting linear features from the data and performs well on linearly separable data. However, if the dataset is linearly indistinguishable and contains a significant amount of non-linear information, then the original feature extraction algorithm may not be effective [33]. To address the limitation of linear feature extraction algorithms in handling nonlinearly separable problems, the KFOLDA feature extraction algorithm was proposed in this study. KFOLDA incorporated a kernel function into the linear feature extraction process. The basic derivation process of KFOLDA is shown in Figure 6.

Let K be the kernel matrix, where $K(x_{i},x_{j})$ is the kernel function value between samples $x_{i}$ and $x_{j}$. In the kernel space, the fuzzy total-scatter matrix and fuzzy between-class scatter matrix are denoted as $K_{fT}$ and $K_{fB}$, respectively. The KFOLDA algorithm is implemented by the following steps.

Firstly, the kernel fuzzy total-scatter matrix $K_{fT}$ is decomposed by SVD as:

(6) $$K_{fT}=G\Sigma S^{T}$$

where $G=[G_{1}G_{2}]$, $G_{1}\in R^{n\times t}$, $\Sigma =\left(\begin{array}{ll} \Sigma_{t} & 0\\ 0 & 0 \end{array}\right)$, $\Sigma_{t}\in R^{t\times t}$, and $t=rank(K_{fT})$.

Secondly, the intermediate matrix B is constructed as:

(7) $$B\;=\;\Sigma_{t}^{-1}G_{1}^{T}K_{fB}$$

where $\Sigma_{t}^{-1}$ is the inverse of the diagonal matrix $\Sigma_{t}$, and $G_{1}^{T}$ is the transpose of $G_{1}$. Then, SVD is performed on B:

(8) $$B\;=\;P_{K}\Lambda O_{K}^{T}$$

where $P_{K}\in R^{t\times t}$ is the left singular vector matrix.

Finally, compute the matrix Y:

(9) $$Y\;=\;G_{1}\Sigma_{t}^{-1}P_{K}$$

and select the first q columns of Y to construct $Y_{q}$, where $q=rank(K_{fB})$. Then, $Y_{q}$ is decomposed by QR decomposition:

(10) $$Y_{q}\;=\;Q_{K}R_{K}$$

where $Q_{K}\in R^{n\times q}$ is the orthogonal matrix and $\left(R_{K}\in R^{q\times q}\right)$ is the upper triangular matrix. The matrix Q is the optimal transformation matrix for feature extraction.

### 2.5. Support Vector Machine

SVM, developed by Vapnik, is a notable classification algorithm that models samples from known categories to categorize unknown samples [34]. SVM is capable of classifying not only linearly separable data sets but also linearly inseparable data sets. For linearly separable data sets, SVM expects to find a maximum separable hyperplane to separate different categories of data. If the dataset is linearly inseparable, the SVM transforms the linearly inseparable dataset into a linearly separable one by using kernel functions to convert the dataset to a higher feature space. The optimal solution is then solved in a linearly separable manner.

SVM with a radial basis function (RBF) kernel function has two critical parameters: the penalty *c* and the function width *g*. The adjustment of parameters holds significant importance in the model’s classification effectiveness. The parameter *c* affects the calculation speed and error of the model and *g* directly determines the learning ability of the model [35,36].

### 2.6. K Nearest Neighbor

KNN is a supervised learning algorithm for classification and regression [37,38]. The fundamental idea of KNN is as follows: given a training dataset with known data points and labels, when a test data point is input, the algorithm compares its characteristics with the matching features in the training data. It then identifies the top K nearest data samples in the training data. The class of the test sample is determined by selecting the class that is most frequently observed among the K nearest data points [39].

In this study, PCA + LDA, PCA + OLDA, PCA + FOLDA and PCA + KFOLDA were respectively used to acquire feature data from the spectral data of the black bean samples. Then, the KNN classifier was employed to construct the classification model of black bean origins. The classification accuracy is influenced by factors such as the size of training and test data points, as well as the parameter K of the KNN classifier.

## 3. Results

### 3.1. Spectral Analysis

In this research, the spectral data of black beans covered a wavelength range of 900 nm to 1700 nm. Figure 5b shows the average spectra of black beans from five different geographical origins. NIR primarily involves the multiplicative and synoptic absorption of the X-H (X = C, N, O) vibrations in groups with hydrogen atoms [40]. By analyzing NIR spectra, it is possible to determine the functional groups, chemical bonds, and molecular structure of organic compounds [41,42]. NIR spectroscopy detects functional groups by analyzing the vibrational frequencies of molecular chemical bonds. There are similarities and differences in the NIR spectral profiles of black beans from different origins, which can be observed across different wavelength ranges. This phenomenon can be attributed to variations in the chemical content of the black bean samples from different origins. For instance, five different black bean samples all have two distinct absorption peaks at 1205 nm and 1485 nm, respectively. The absorption peak at 1205 nm is associated with the C-H stretching vibration and the second overtone of the CH2 and CH3 bonds [43]. The black bean spectra showed a sharp increase in absorbance from 1300 nm to 1400 nm, which can be attributed to the C-H stretching vibration of the CH3 and CH2 bonds and the O-H stretching, the first overtone [44]. The C-H bonds mainly derive from lipids, carbohydrates and protein skeletons in black bean samples; the O-H bonds come from water, carbohydrates and phenolic substances. As shown in Figure 5f, the entire spectrum peaks at 1485 nm, the first harmonic of the O-H stretching vibration, which can be attributed to the presence of water or the N-H stretch second overtone in amides and aromatic amines. It represents a superimposed composite signal within 1460–1520 nm: apart from the O-H vibration of water molecules, the first overtone of alcoholic-OH from carbohydrates, the first overtone of phenolic-OH from phenolic compounds, as well as the second overtone of N-H stretch in amides and aromatic amines all overlap in this spectral region [45,46]. The absorbance of various black bean samples in the range of 900–1700 nm indicates that Sichuan has a significantly higher absorbance spectrum than the other four categories of black bean varieties. The spectral absorbance of the five types of black beans begins to differ after 1400 nm. This spectral information can reveal subtle differences between the different origins of black bean samples, highlighting the importance of NIR spectral data in providing a necessary foundation for the categorization of different origins of black bean samples.

### 3.2. Spectral Preprocessing

As illustrated in Figure 5a–e, five pre-processing algorithms, namely SG, SNV, MSC, MC and 1st D, were employed to experimentally process the spectral data of the black bean samples. The aforementioned algorithms were employed to remove extraneous noise and irrelevant data points, while simultaneously improving the precision of the classification model. Table 1 shows the identification results of four feature extraction algorithms coupled with pre-processing algorithms on the test set (KNN as classifier, K = 7). It can be seen that the SG algorithm is significantly better than the other four pre-processing algorithms. This indicates that the original spectral data are mixed with a lot of disruptive noise, which may be affected by the external environment during the measurement. After the SG preprocessing, the experiment performed PCA + LDA, PCA + FST, PCA + UDT, PCA + OLDA, PCA + FOLDA and PCA + KFOLDA to extract the features of the spectral data of black bean samples. The accuracy of the six classification models will be presented separately below.

### 3.3. Classification with PCA + LDA

**Figure 7:**
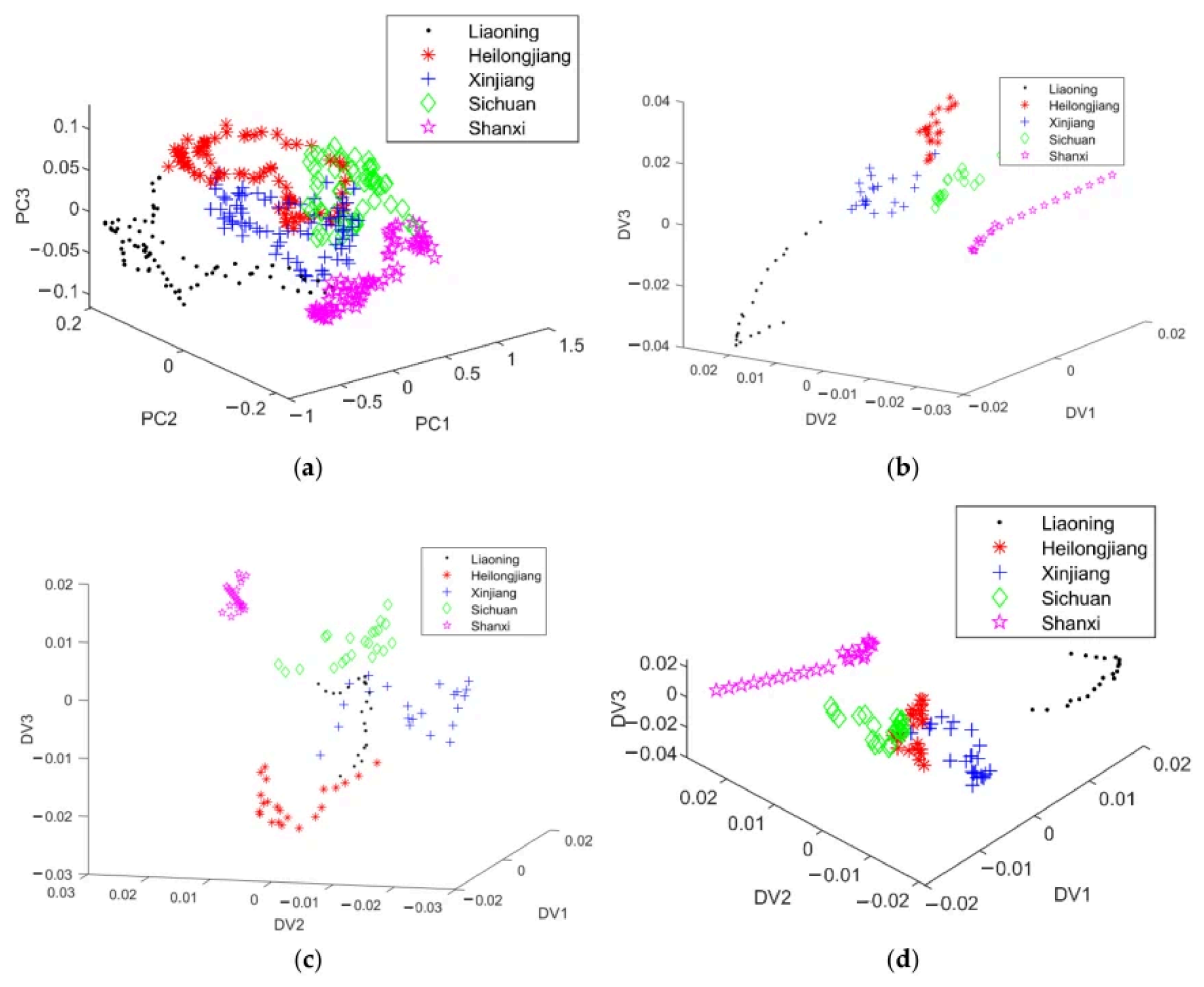
The 3D data distribution by feature extraction methods: (**a**) PCA, (**b**) FST, (**c**) UDT, and (**d**) OLDA.

The comparison of model performance in this study can be evaluated by the classification accuracy of different models. The spectral data of the black bean samples formed a 400 × 400 matrix as the 400-dimensional data. At this stage, the NIR spectra were incorporated into a significant amount of redundant data that affected the classification accuracy. Therefore, data dimensionality reduction was necessary to eliminate redundant information and improve the model performance. This study used PCA to compress the high-dimensional data. The number of principal components (PCs) has a significant impact on the model’s classification accuracy, and the cumulative contribution ratio (CCR) is usually used as the condition for selecting the number of PCs [47]. The CCR of the first 10 PCs of PCA was as high as 99.96%, so the first 10 PCs of PCA were selected to replace the high-dimensional data, which not only reduced the loss of data information but also allowed for data dimensionality compression. Figure 7a illustrates the distribution of black bean data after PCA dimensionality reduction. Evidently, PCA did not perform well in identifying the black bean samples. To better extract feature information, the data dimensions were compressed again using LDA. A total of 58 samples were selected from each type of black bean sample as the training data, and the rest served as the test data. The number of discriminant vectors was 4 (*c* − 1), and then the 10-dimensional compressed data was transformed into the four-dimensional data after the feature transformation of LDA. PCA + LDA could lower the dimensions of high-dimensional data and further extract feature information, thus achieving a classification accuracy of 80.9%.

### 3.4. Classification with PCA + FST

This study employed FST as another feature extraction method for comparison. FST processed the 10-dimensional training samples to obtain five optimal discriminative vectors. The 10-dimensional data from the test set were projected into the orthogonal vector space of FST. Figure 7b shows the distribution of data points after FST processing. We observed that the data distribution of black beans in Liaoning and Shanxi was better separated. However, data from the other three regions exhibited significant overlap. The classification accuracy of PCA + FST was 84.5%, indicating that there is still room for improvement.

### 3.5. Classification with PCA + UDT

To address the issue of data correlation in FST, we used UDT as a better feature extraction tool. The 10-dimensional training samples were processed to obtain four optimal discriminative vectors by UDT, providing a better extraction of feature information. Figure 7c shows the arrangement of data points after UDT processing. A great separation result can be observed between Shanxi, Heilongjiang and Sichuan. However, there is evident overlap between the samples from Liaoning and Xinjiang, which may affect the classification result. Finally, compared to PCA + FST, PCA + UDT achieved a higher classification rate of 85.5%.

### 3.6. Classification with PCA + OLDA

To overcome the SSS problem arising from LDA, this study employed OLDA for feature extraction from the sample data. OLDA processed the 10-dimensional training samples to obtain four optimal discriminative vectors. The 10-dimensional data of the remaining 110 test samples were projected onto the four discriminant vectors of OLDA, achieving the purpose of effective extraction of feature information. Figure 7d shows the arrangement of data points after OLDA processing. We observed that the data distribution of black beans in Liaoning, Sichuan, and Shanxi exhibited better separation, whereas the data distribution in Heilongjiang and Xinjiang had more overlapping regions. As a result, the classification accuracy of PCA + OLDA was only 83.6%.

### 3.7. Classification with PCA + FOLDA

PCA + LDA and PCA + OLDA did not yield a higher classification accuracy. Therefore, the identification information from the NIR spectral data was extracted by FOLDA after PCA. The following parameters were configured: the fuzzy weight index *m* = 1.6, the count of sample categories *c* = 5, and the initial clustering centers of FOLDA:

$$V_{\mathrm{FOLDA}}^{\left(0\right)}=\left[\begin{array}{l} v_{1}^{\left(0\right)}\\ v_{2}^{\left(0\right)}\\ v_{3}^{\left(0\right)}\\ v_{4}^{\left(0\right)}\\ v_{5}^{\left(0\right)} \end{array}\right]=\left[\begin{array}{cccccccccc} -0.6108 & 0.0568 & -0.0440 & 0.0200 & -0.0044 & 0.0023 & -0.0026 & 0.0016 & 0.0013 & 0.0001\\ 0.1817 & 0.0676 & 0.0320 & -0.0227 & -0.0045 & -0.0007 & -0.0063 & 0.0044 & -0.0001 & -0.0007\\ 0.1605 & 0.0067 & -0.0107 & 0.0123 & 0.0187 & -0.0053 & 0.0043 & 0.0018 & -0.0003 & -0.0001\\ 0.6599 & 0.0187 & 0.0178 & 0.0097 & -0.0108 & 0.0003 & 0.0019 & -0.0045 & -0.0001 & -0.0010\\ -0.2746 & -0.1998 & -0.0168 & -0.0197 & -0.0018 & 0.0023 & 0.0006 & -0.0039 & 0.0011 & -0.0005 \end{array}\right]$$

**Figure 8:**
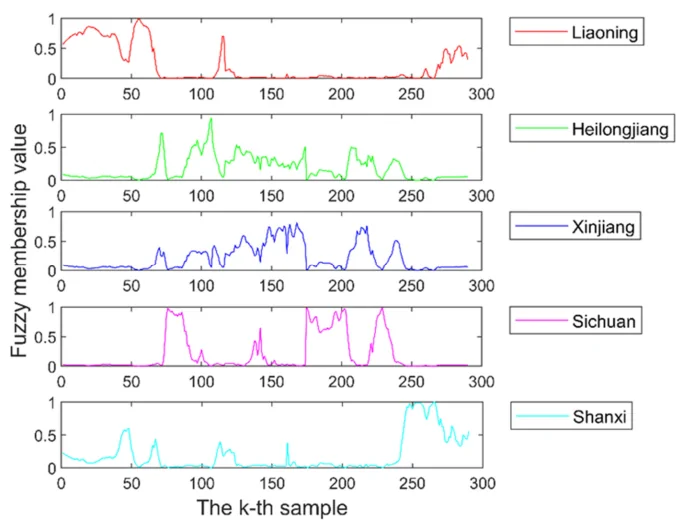
Initial fuzzy membership values of each black bean sample.

The initial values of fuzzy membership in FOLDA are shown in Figure 8, where the training set and fuzzy membership values are represented by the abscissa and ordinate, respectively. The small icons on the right represent different origins of black beans, namely Liaoning, Heilongjiang, Xinjiang, Sichuan and Shanxi. The degree to which each black bean sample belonged to each variety could be expressed through the fuzzy membership function. Samples were considered to belong to the breed when their corresponding fuzzy membership value exceeded 0.5. We confirmed that the *i*th sample belonged to *j*th class when the value of the fuzzy affiliation of the *i*th sample *u_ij_* was the largest in *j*th class. Taking the first subplot in Figure 8 as an example, it illustrates the membership degrees of 290 samples belonging to the Liaoning category, with values ranging between 0 and 1. As shown in Figure 8, the membership degrees of Liaoning samples within their own category predominantly approach 1, while their membership degrees in other categories are nearly 0. However, the fuzzy membership degrees of Heilongjiang and Heilongjiang samples exhibit partial overlap within the same interval. This observation aligns with the analysis of the average spectral plots, where the spectral curves of Heilongjiang and Heilongjiang black bean samples show a high degree of similarity, indicating that their spectral features are nearly indistinguishable. Such feature overlap may lead to misclassification when the model attempts to differentiate between Heilongjiang and Heilongjiang samples, thereby limiting further improvements in classification performance.

**Figure 9:**
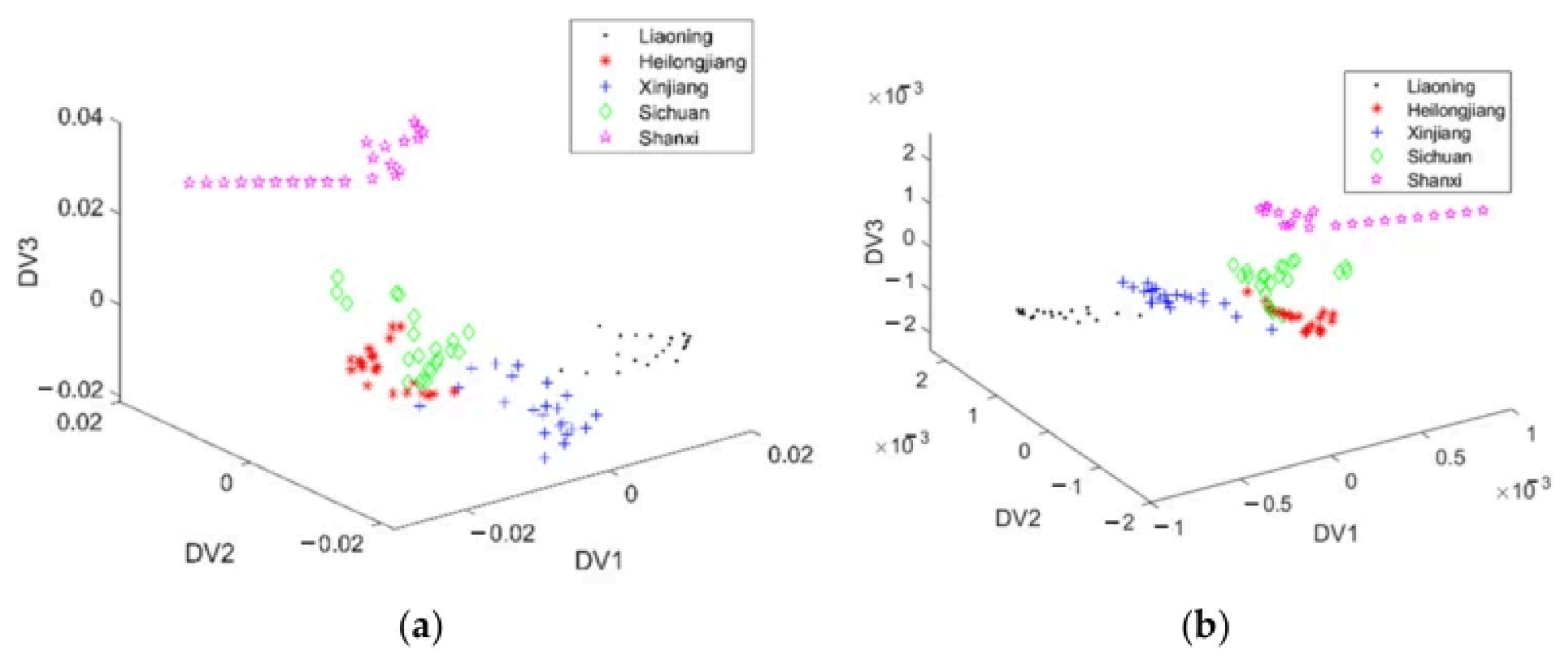
The 3D data distribution by feature extraction methods: (**a**) PCA + FOLDA; (**b**) PCA + KFOLDA.

Figure 9a illustrates the 3D scores plot of FOLDA extracting discriminative information from black bean test samples. It can be seen from Figure 9a that the data from Liaoning and Heilongjiang were relatively far apart, and their data could be accurately distinguished. The number of overlapping data from Sichuan and Xinjiang was relatively high, so achieving an accurate classification is somewhat difficult. From the results, Figure 9a showed a better distribution for classification than Figure 7d. The combination of PCA and FOLDA improved the model’s classification accuracy, achieving a classification accuracy of 86.4%.

### 3.8. Classification with PCA + KFOLDA

One way to effectively address the issue of nonlinearity in separability is by incorporating kernel functions into the feature extraction algorithm. This method will also be validated in the current experiment. This study introduced linear kernel functions into FOLDA and proposed the KFOLDA, a nonlinear feature extraction algorithm. The following parameters were preset: the fuzzy weight index *m* = 1.6, the number of sample categories *c* = 5, and the initial clustering centers and initial values of fuzzy membership in KFOLDA were consistent with FOLDA. Firstly, a kernel function transformed the spectral data into a kernel feature space. After calculation, the transformation matrix of KFOLDA was:

$$W={\left[\begin{array}{cccc} -0.0937 & 0.0042 & -0.0387 & -0.0151\\ -0.0847 & -0.0044 & -0.0283 & -0.0127\\ \vdots & \vdots & \vdots & \vdots \\ 0.0334 & -0.0101 & 0.0285 & 0.0454 \end{array}\right]}_{290\times 4}$$

Based on the transformation matrix *W*, the 10-dimensional black bean data were mapped to four discriminant vectors: DV1, DV2, DV3, and DV4 of KFOLDA. Figure 9b shows the 3D score of the test samples after PCA + KFOLDA feature extraction. By comparing Figure 7b and Figure 9b, it can be seen that the data separation of different origins of black beans in Figure 9b has been significantly improved. The samples from LN (Liaoning), XJ (Xinjiang), and SX (Shanxi) exhibit no overlapping regions, with data points within each category being more densely clustered and the boundaries between categories becoming more distinct. Only a small number of samples from HLJ (Heilongjiang) and SC (Sichuan) show overlapping phenomena. This distribution provides a more favorable data structure for subsequent classification tasks. Therefore, the kernel function solved the nonlinearly separable problem and improved the data distribution for classification.

**Figure 10:**
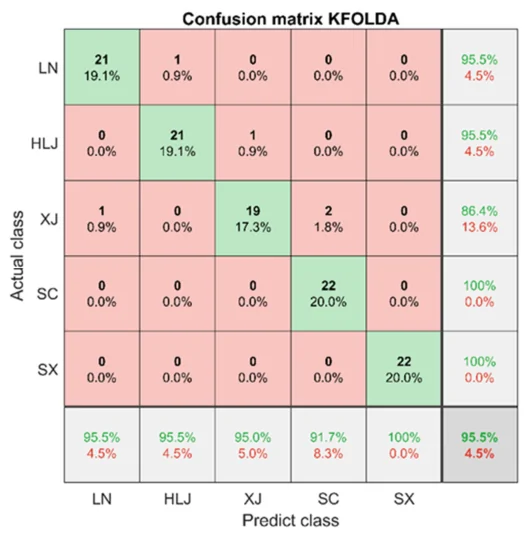
Classification outcome of PCA + KFOLDA combined with KNN on test set data.

Figure 10 illustrates the classification results of the PCA + KFOLDA algorithm using a KNN classifier. The KFOLDA confusion matrix provides a clear visualization of the classification outcomes for black beans from different geographical origins. In the matrix, rows represent predicted classes, while columns correspond to true classes. The diagonal cells indicate the number of correctly classified samples and their respective classification accuracies, whereas the off-diagonal cells display the distribution of misclassified samples. Additionally, the rightmost column lists the classification accuracy for each class, the bottom row shows the recall rates, and the gray cell at the bottom-right corner denotes the overall classification accuracy of the KFOLDA algorithm. From Figure 10, it is evident that all test samples from SC and SX are correctly classified, achieving a high accuracy of 100%. One sample of HLJ and one sample of LN were misclassified, while three samples from XJ were incorrectly labeled. These results demonstrate that the KFOLDA-based discrimination model exhibits higher classification accuracy, effectively distinguishing black bean samples from different geographical origins. Finally, PCA + KFOLDA achieved a classification accuracy of 95.5%.

### 3.9. Comparison with KNN and SVM

**Figure 11:**
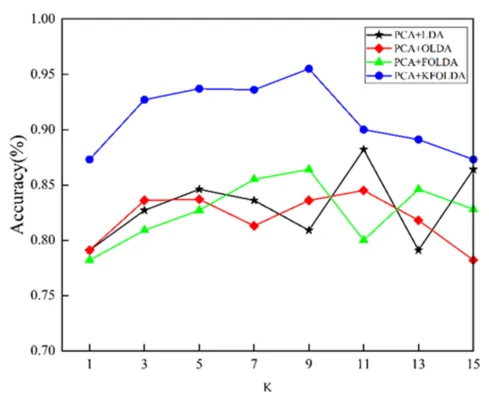
Classification accuracy of four feature extraction algorithms under different K.

**Figure 12:**
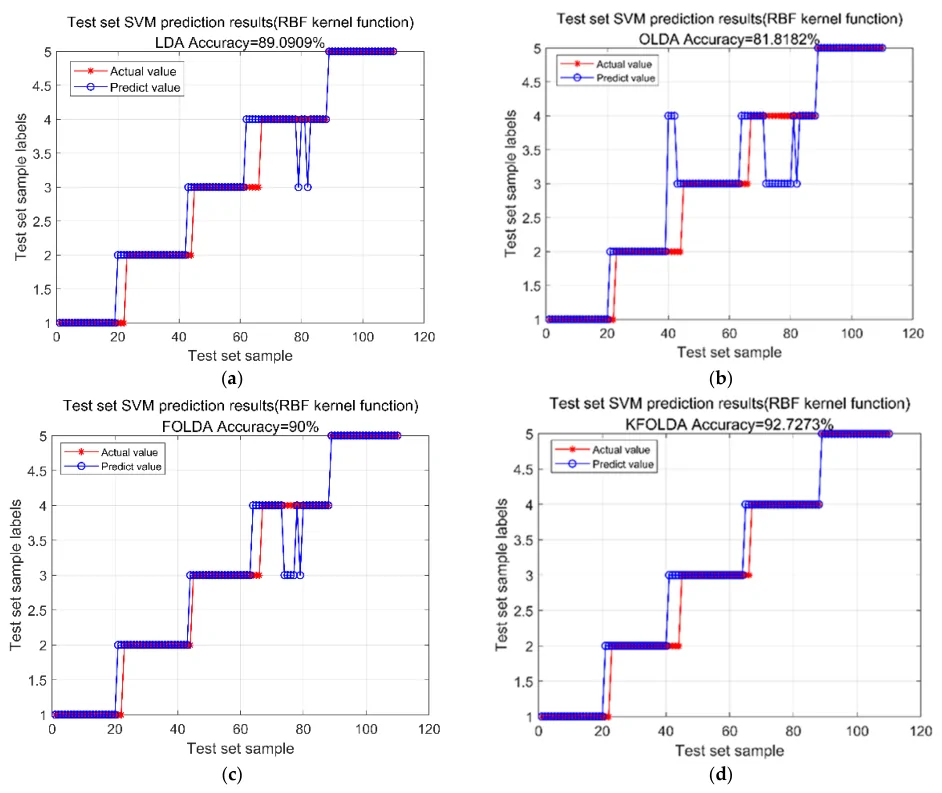
Classification of four feature extraction algorithms under SVM: (**a**) PCA + LDA, (**b**) PCA + OLDA, (**c**) PCA + FOLDA, and (**d**) PCA + KFOLDA.

In order to reflect the performance of the black bean variety identification model, this study employed two different classification models, namely KNN and SVM, to classify black bean samples and compare the final results. The parameter K has a significant impact on the accuracy of the KNN algorithm. Therefore, in this study, four different feature extraction algorithms were performed to obtain the model classification accuracies corresponding to different K values (K = 1, 3, 5, 7, 9, 11, 13, 15). To reflect the impact of parameter adjustment on performance, this experiment ensured that other parameters remained unchanged (especially the number of samples in the training set and test set). By changing the parameter K, the comparison results were illustrated in Figure 11. From Figure 11, it was evident that when the value of K was small, the accuracy of PCA + FOLDA was lower compared to that of PCA + LDA and PCA + OLDA. This could be attributed to the possibility of overfitting, which occurs when the K value is too small. On the other hand, if the value of K is too large, the model may become too simple, which could potentially lead to an increase in classification errors. The classification models consisting of PCA + KFOLDA and PCA + FOLDA algorithms worked best when the value of K was in the middle range. Furthermore, the experiment utilized SVM as another classification algorithm to distinguish different origins of black beans. The kernel function employed for the SVM was the RBF, with the penalty and function width ranging from 2-2 to 22, with a step size of 0.2 [48,49]. The experiment was conducted using five-fold cross-validation to find the optimal parameters that would achieve the best performance of the model. Figure 12 presents the classification results of four discriminant analysis algorithms using SVM.

Comparing SVM with KNN, the results are displayed in Table 2. The results indicated that using PCA + FOLDA in combination with SVM led to higher classification accuracy for black bean samples. This could be attributed to the effectiveness of the RBF kernel function used in SVM, which is known to perform well when dealing with nonlinear problems. However, from a comprehensive point of view, KNN had a better classification performance on different origins of black beans. The results showed that FOLDA and KFOLDA extracted more effective information from the NIR spectra of black bean samples, achieving classification accuracies of 86.4% and 95.5%, respectively.

## 4. Discussion

### 4.1. Parameter and Performance Analysis of FOLDA and KFOLDA

In this study, the best classification model could be tested by varying the size of training and test samples. With other parameters held constant, Table 3 shows the classification accuracy of the black bean variety for different numbers of training data. It can be seen from Table 3 that when the number of training samples was 58 and the number of test samples was 22, the accuracy of PCA + FOLDA achieved 86.4% and the accuracy of PCA + KFOLDA reached as high as 95.5%.

**Figure 13:**
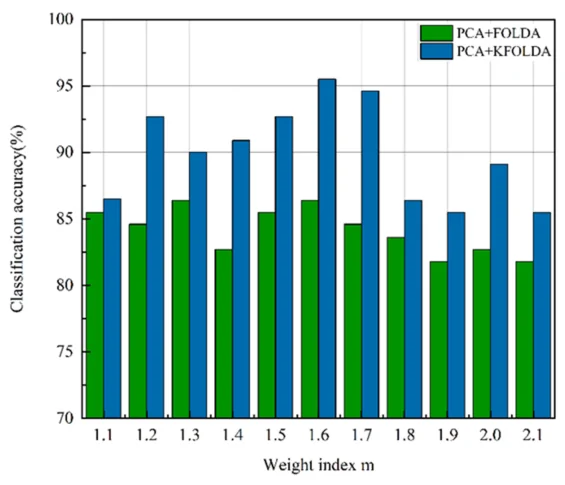
Classification accuracies of PCA + FOLDA and PCA + KFOLDA in different weight indices *m*.

The fuzzy membership function was introduced in both FOLDA and KFOLDA, and the weight index *m* in the fuzzy membership function played a crucial role in the results of the fuzzy feature extraction algorithm. When the value of *m* was too small, the fuzzy feature extraction algorithm would not be able to effectively distinguish overlapping data points. When the value of *m* was too large, the data samples belonging to different types became very blurred, resulting in a decrease in classification accuracy. Selecting an appropriate weight index *m* is essential for achieving the best classification results. From Figure 13, we could find that the classification accuracy of FOLDA and KFOLDA increased with the increase in the index *m*, and when *m* was 1.6, the accuracy of classification was at its maximum point. Afterwards, as *m* continued to increase, the classification accuracy indicated a downward trend. Therefore, this study chose the optimal weight index *m* value of 1.6.

In the analysis of the KFOLDA algorithm, this study chose the linear kernel function as the object combined with FOLDA. The operation in the linear kernel function involves the inner product of two vectors and does not require parameter optimization, which greatly reduces computation time and cost. In this experiment, the number of attributes in the black bean dataset was relatively small after dimensionality reduction and feature extraction. Therefore, the linear kernel was found to be more suitable for handling this situation [50]. In previous studies, many scholars combined fuzzy set theory or kernel functions with different classifiers to train a classification model with stronger generalization ability and higher classification accuracy [51,52]. This article explored this from another perspective by combining fuzzy set theory with feature extraction algorithms, and further introducing kernel functions into fuzzy set theory to extract more identification information from NIR data of black bean samples. The more discriminant information is retained in the data, the more accurate the classification is obtained in the model. FOLDA and KFOLDA are two novel feature extraction algorithms with different applications. FOLDA can produce good classification results when the dataset has many linear features. On the other hand, KFOLDA can transform datasets with non-linear features into linear features for classification, thereby achieving good performance.

Compared with traditional OLDA, FOLDA can extract more effective discriminative information from overlapping black bean data, while KFOLDA further enhances the classification ability for nonlinear data by introducing kernel functions. In this study, the extracted features of black bean data were used as input, and the KNN classifier was employed to analyze the geographical origin of the black beans. Table 4 presents the classification performance of different models on the test set, including classification accuracy, sensitivity, and specificity. As shown in Table 4, FOLDA-KNN and KFOLDA-KNN achieved the best performance across all three metrics, significantly outperforming other methods. This further demonstrates the advantages of combining these two feature extraction methods with the KNN classifier in identifying the geographical origins of black beans.

### 4.2. Feature Extraction Mechanism Comparison

In the present study, several discriminant feature extraction algorithms, including LDA, OLDA, FOLDA and KFOLDA, were constructed for geographical origin identification of black beans based on portable NIR spectral data. Traditional LDA suffers from the well-known small sample size (SSS) problem, resulting in an unstable transformation matrix and limited capability to extract discriminative information from overlapping spectral samples, which only yielded 80.9% classification accuracy in combination with KNN. As an improved orthogonal discriminant method, OLDA alleviates the matrix singularity issue of LDA by imposing orthogonality constraints on discriminant vectors, yet it still treats each sample with hard category assignment and ignores sample fuzziness caused by spectral overlap, so its classification accuracy merely reached 83.6%. By introducing fuzzy membership into OLDA, FOLDA enables soft partitioning for ambiguous samples and enhances the capacity to handle noisy and overlapping spectral data, and the FOLDA-KNN model achieved an accuracy of 86.4%. Nevertheless, FOLDA is essentially a linear feature extraction approach and cannot fully mine intrinsic nonlinear relationships embedded in high-dimensional NIR spectra. By further embedding the linear kernel into FOLDA, the proposed KFOLDA effectively maps original spectral features into kernel space to extract nonlinear discriminant information. Consequently, the KFOLDA-KNN model obtained the highest accuracy of 95.5%, and it also exhibited superior performance in terms of specificity and sensitivity among all compared linear discriminant methods.

When compared with existing related work that developed the UDT-XGBoost framework for black bean origin discrimination [53], both studies focused on portable NIR traceability for black beans from five producing regions and adopted SG as one of the core spectral preprocessing methods. The UDT-XGBoost combination realized 96% average accuracy, benefiting from the powerful nonlinear fitting ability of the ensemble tree classifier XGBoost. However, the excellent performance of UDT-XGBoost heavily depends on careful hyperparameter tuning for tree depth, subsample ratio and regularization coefficients, which increases model complexity and computational overhead. Different from the strategy of coupling feature extraction with an ensemble boosting classifier in the literature, this work innovatively integrates fuzzy set and kernel mechanisms within the feature extraction stage itself. The KFOLDA algorithm completes nonlinear feature transformation before inputting features into a simple KNN classifier; it avoids tedious hyperparameter optimization for complex ensemble models and maintains relatively low computational cost. In addition, fuzzy membership inside FOLDA/KFOLDA can quantitatively characterize the category ambiguity of confusing samples, which provides interpretable intermediate information for analyzing misclassification phenomena, while UDT outputs uncorrelated features without such fuzzy quantitative description for sample uncertainty. Admittedly, the accuracy of KFOLDA-KNN is slightly lower than the optimal result of UDT-XGBoost, but KFOLDA-KNN achieves competitive overall performance without relying on complicated ensemble classifiers, which is more friendly for embedded deployment on portable NIR detection terminals in practical on-site scenarios.

This study only employs a linear kernel, so future experiments may explore alternative kernel functions. Moreover, swarm intelligence-based optimisation algorithms can be utilised to determine the optimal parameter combination for kernel-based models and further enhance model performance.

## 5. Conclusions

In this study, portable NIR spectroscopy combined with novel fuzzy feature extraction algorithms was developed to realize rapid and non-destructive geographical origin identification of black beans. Raw NIR spectra acquired from black bean samples of five typical producing regions were preprocessed by the SG algorithm to mitigate spectral noise and light scattering interference. This work proposes two feature extraction approaches, namely fuzzy orthogonal linear discriminant analysis (FOLDA) and its kernel extension (KFOLDA). By embedding fuzzy set theory into orthogonal linear discriminant analysis, FOLDA assigns fuzzy membership values to individual samples, which strengthens the capability to handle overlapping and noisy spectral data and fully characterizes sample diversity. On this basis, kernel fuzzy orthogonal linear discriminant analysis (KFOLDA) further introduces a linear kernel function, mapping original spectral data into a high-dimensional kernel space so as to extract the intrinsic nonlinear discriminant information hidden within NIR spectra.

Experimental results demonstrate that the PCA-FOLDA model achieves a classification accuracy of 86.4%, whereas PCA-KFOLDA yields a much higher accuracy of 95.5% with superior sensitivity and specificity metrics. Compared with conventional discriminant methods including LDA, OLDA, FST and UDT, the proposed KFOLDA effectively overcomes the limitations of linear algorithms when coping with non-linearly separable spectral datasets. Without relying on complex ensemble classifiers, KFOLDA combined with a simple KNN classifier obtains competitive classification performance, which renders it suitable for lightweight deployment on portable NIR analytical terminals.

From an application perspective, the proposed framework combining a portable NIR spectrometer with FOLDA/KFOLDA exhibits promising practical value for black bean origin traceability. It provides a feasible analytical scheme for rapid origin screening of black bean commodities on-site, helping curb origin adulteration in the bean product market and safeguard consumer interests. Nevertheless, several drawbacks remain in the current investigation. First, the present study only collected black bean samples from five main production zones; samples with more complex genetic backgrounds and growth conditions should be supplemented to further validate model generalization. Second, only the linear kernel was adopted for KFOLDA; other kernel types and multi-kernel combination strategies were not systematically explored. Third, all experiments were carried out under laboratory conditions, and practical field validation under complex real-world scenarios such as instrument drift and environmental disturbance is absent.

Future research will expand the sample set covering more geographical origins, screen optimal kernel function configurations, and implement field industrial tests. Further efforts will also focus on optimizing model computation overhead to facilitate embedded implementation for on-site detection. Overall, this research validates that portable NIR spectroscopy coupled with KFOLDA establishes an effective and efficient framework for black bean geographical origin discrimination, offering a potential strategy for rapid, non-destructive quality traceability of legume food.

## Figures and Tables

**Table 1:** Classification accuracies of each model under different preprocessing algorithms.

	SG	MSC	SNV	MC	1st D
PCA + LDA	80.9%	56.4%	51.8%	57.3%	69.1%
PCA + FST	84.5%	60.1%	52.7%	53.6%	61.8%
PCA + UDT	85.5%	61.8%	54.5%	51.8%	63.7%
PCA + OLDA	83.6%	65.5%	60.0%	56.4%	60.9%
PCA + FOLDA	86.4%	62.7%	59.1%	52.7%	66.4%
PCA + KFOLDA	95.5%	57.3%	60.9%	56.4%	62.7%

**Table 2:** Classification accuracies of four feature extraction algorithms under KNN and SVM.

	PCA + LDA	PCA + FST	PCA + UDT	PCA + OLDA	PCA + FOLDA	PCA + KFOLDA
KNN	80.9%	84.5%	85.5%	83.6%	86.4%	95.5%
SVM	89.1%	86.4%	88.2%	81.8%	90.0%	92.7%

**Table 3:** Identification accuracies with different numbers of training data and test data.

n_training	n_test	PCA + LDA	PCA + FST	PCA + UDT	PCA + OLDA	PCA + FOLDA	PCA + KFOLDA
270	130	80.8%	83.1%	84.6%	83.1%	88.5%	87.7%
280	120	80.0%	80.8%	81.7%	83.3%	83.3%	90.1%
290	110	80.9%	84.5%	85.5%	83.6%	86.4%	95.5%
300	100	89.0%	88.0%	90.0%	81.0%	83.0%	92.0%
310	90	87.8%	85.6%	88.9%	83.3%	78.9%	91.1%

**Table 4:** Accuracy, specificity, and sensitivity of different models on the test set.

Models	Accuracy	Specificity	Sensitivity
PCA + LDA-KNN	80.9%	80.9%	95.2%
PCA + OLDA-KNN	83.6%	83.6%	95.9%
PCA + FOLDA-KNN	86.4%	86.3%	96.6%
PCA + KFOLDA-KNN	95.5%	95.5%	98.9%

## Data Availability

Data and the model implementation will be made available on request from the corresponding author.

## References

[1] Meenu M., Chen P., Mradula M., Chang S.K., Xu B. (2023). New insights into chemical compositions and health-promoting effects of black beans (*Phaseolus vulgaris* L.). Food Front..

[2] Zhang R.F., Zhang F.X., Zhang M.W., Wei Z.C., Yang C.Y., Zhang Y., Tang X.J., Deng Y.Y., Chi J.W. (2011). Phenolic composition and antioxidant activity in seed coats of 60 Chinese black soybean (*Glycine max* L. Merr.) varieties. J. Agric. Food Chem..

[3] He L., Hu Q., Zhang J., Xing R., Zhao Y., Yu N., Chen Y. (2023). An integrated untargeted metabolomic approach reveals the quality characteristics of black soybeans from different geographical origins in China. Food Res. Int..

[4] Dai C., Sun J., Huang X., Zhang X., Tian X., Wang W., Luan Y. (2023). Application of hyperspectral imaging as a nondestructive technology for identifying tomato maturity and quantitatively predicting lycopene content. Foods.

[5] Sun J., Yang F., Cheng J., Wang S., Fu L. (2024). Nondestructive identification of soybean protein in minced chicken meat based on hyperspectral imaging and VGG16-SVM. J. Food Compos. Anal..

[6] Mendoza F.A., Kelly J.D., Cichy K.A. (2017). Automated prediction of sensory scores for color and appearance in canned black beans (*Phaseolus vulgaris* L.) using machine vision. Int. J. Food Prop..

[7] Sun J., Zhou L., He Y., Gui L., Cheng Y., Xie M., Liu S., Shen Z., Li Y. (2025). Comprehensive evaluation of processing suitability of different tea cultivars for Huangshan Maofeng green tea. J. Food Compos. Anal..

[8] Kılıç K., Boyacı İ.H., Köksel H., Küsmenoğlu İ. (2007). A classification system for beans using computer vision system and artificial neural networks. J. Food Eng..

[9] Pérez-Martín L., Bustamante-Rangel M., Delgado-Zamarreño M.M. (2017). Classification of lentils, chickpeas and beans based on their isoflavone content. Food Anal. Methods.

[10] Fan G., Beta T. (2017). Discrimination of geographical origin of Napirira bean (*Phaseolus vulgaris* L.) based on phenolic profiles and antioxidant activity. J. Food Compos. Anal..

[11] Tahir H.E., Xiaobo Z., Tinting S., Jiyong S., Mariod A.A. (2016). Near-infrared (NIR) spectroscopy for rapid measurement of antioxidant properties and discrimination of Sudanese honeys from different botanical origin. Food Anal. Methods.

[12] Wang J., Guo Z., Zou C., Jiang S., El-Seedi H.R., Zou X. (2022). General model of multi-quality detection for apple from different origins by Vis/NIR transmittance spectroscopy. J. Food Meas. Charact..

[13] Zhang H., Jiang H., Liu G., Mei C., Huang Y. (2017). Identification of radix puerariae starch from different geographical origins by FT-NIR spectroscopy. Int. J. Food Prop..

[14] Sun J., Jiang S., Mao H., Wu X., Li Q. (2016). Classification of black beans using visible and near infrared hyperspectral imaging. Int. J. Food Prop..

[15] Correia R.M., Tosato F., Domingos E., Rodrigues R.R., Aquino L.F.M., Filgueiras P.R., Romao W. (2018). Portable near infrared spectroscopy applied to quality control of Brazilian coffee. Talanta.

[16] Lu J., Ma G., Zhang G. (2024). Fuzzy machine learning: A comprehensive framework and systematic review. IEEE Trans. Fuzzy Syst..

[17] Saatchi R. (2024). Fuzzy Logic Concepts, Developments and Implementation. Information.

[18] Qi Z., Wu X., Yang Y., Wu B., Fu H. (2022). Discrimination of the red jujube varieties using a portable NIR spectrometer and fuzzy improved linear discriminant analysis. Foods.

[19] Abe S. (2015). Fuzzy support vector machines for multilabel classification. Pattern Recognit..

[20] Ye J., Yu B. (2005). Characterization of a family of algorithms for generalized discriminant analysis on undersampled problems. J. Mach. Learn. Res..

[21] Hofmann T., Schölkopf B., Smola A.J. (2008). Kernel methods in machine learning. Ann. Stat..

[22] Al-Dabagh M.Z.N., Alhabib M.M., Al-Mukhtar F.H. (2018). Face recognition system based on kernel discriminant analysis, k-nearest neighbor and support vector machine. Int. J. Res. Eng..

[23] Jiang J., Huang L., Li H., Xiao L. (2016). Hyperspectral image supervised classification via multi-view nuclear norm based 2D PCA feature extraction and kernel ELM. Proc. Int. Geosci. Remote Sens. Symp..

[24] Plans M., Simó J., Casañas F., Sabaté J., Rodriguez-Saona L. (2013). Characterization of common beans (*Phaseolus vulgaris* L.) by infrared spectroscopy: Comparison of MIR, FT-NIR and dispersive NIR using portable and benchtop instruments. Food Res. Int..

[25] Miloš B., Bensa A., Japundžić-Palenkić B. (2022). Evaluation of Vis-NIR preprocessing combined with PLS regression for estimation soil organic carbon, cation exchange capacity and clay from eastern Croatia. Geoderma Reg..

[26] Shi X., Song J., Wang H., Lv X., Zhu Y., Zhang W., Zeng L. (2023). Improving soil organic matter estimation accuracy by combining optimal spectral preprocessing and feature selection methods based on pXRF and vis-NIR data fusion. Geoderma.

[27] Lei F., Yang Y., Zhang J., Zhong J., Yao L., Chen J., Pan T. (2019). Window optimisation PMSC–PLS with applications to NIR spectroscopic analyses. Chemom. Intell. Lab. Syst..

[28] Yang Z., Xiao H., Zhang L., Feng D., Zhang F., Jiang M., Sui Q., Jia L. (2020). Fast determination of oxides content in cement raw meal using NIR spectroscopy combined with synergy interval partial least square and different preprocessing methods. Measurement.

[29] Mutlag W.K., Ali S.K., Aydam Z.M., Taher B.H. (2020). Feature extraction methods: A review. J. Phys. Conf. Ser..

[30] Liang Y., Lin H., Kang W., Shao X., Cai J., Li H., Chen Q. (2023). Application of colorimetric sensor array coupled with machine-learning approaches for the discrimination of grains based on freshness. J. Sci. Food Agric..

[31] Foley D.H., Sammon J.W. (1975). An optimal set of discriminant vectors. IEEE Trans. Comput..

[32] Jin Z., Yang J., Hu Z., Lou Z. (2001). Face recognition based on the uncorrelated discriminant transformation. Pattern Recognit..

[33] Campbell C. (2002). Kernel methods: A survey of current techniques. Neurocomputing.

[34] Li H., Liang H., Miao C., Cao L., Feng X., Tang C., Li E. (2016). Novel ECG signal classification based on KICA nonlinear feature extraction. Circuits Syst. Signal Process..

[35] Cortes C., Vapnik V. (1995). Support-vector networks. Mach. Learn..

[36] Wang S., Sun J., Fu L., Xu M., Tang N., Cao Y., Jing J. (2022). Identification of red jujube varieties based on hyperspectral imaging technology combined with CARS-IRIV and SSA-SVM. J. Food Process..

[37] Eslamian S.S., Gohari S.A., Biabanaki M., Malekian R. (2008). Estimation of monthly pan evaporation using artificial neural networks and support vector machines. J. Appl. Sci..

[38] Li H., Wu P., Dai J., Pan T., Holmes M., Chen T., Zou X. (2023). Discriminating compounds identification based on the innovative sparse representation chemometrics to assess the quality of Maofeng tea. J. Sci. Food Agric..

[39] Zhang S., Li X., Zong M., Zhu X., Cheng D. (2017). Learning k for KNN classification. ACM Trans. Intell. Syst. Technol..

[40] Cunningham P., Delany S.J. (2021). K-nearest neighbour classifiers—A tutorial. ACM Comput. Surv..

[41] Carbas B., Machado N., Oppolzer D., Ferreira L., Brites C., Rosa E.A., Barros A.I. (2020). Comparison of near-infrared (NIR) and mid-infrared (MIR) spectroscopy for the determination of nutritional and antinutritional parameters in common beans. Food Chem..

[42] Fernández-Espinosa A.J. (2016). Combining PLS regression with portable NIR spectroscopy to on-line monitor quality parameters in intact olives for determining optimal harvesting time. Talanta.

[43] Manley M. (2014). Near-infrared spectroscopy and hyperspectral imaging: Non-destructive analysis of biological materials. Chem. Soc. Rev..

[44] Meenu M., Kamboj U., Sharma A., Guha P., Mishra S. (2016). Green method for determination of phenolic compounds in mung bean (*Vigna radiata* L.) based on near-infrared spectroscopy and chemometrics. Int. J. Food Sci. Technol..

[45] Plans M., Simo J., Casanas F., Sabate J. (2012). Near-infrared spectroscopy analysis of seed coats of common beans (*Phaseolus vulgaris* L.): A potential tool for breeding and quality evaluation. J. Agric. Food Chem..

[46] Cheng Y., Wang Y., Leng T., Zhu L., Jing Y., Xie J., Xu Q., Chen Y. (2022). Feasibility study on prediction of the grain mixtures for black sesame paste recipe with different chemometric methods. LWT.

[47] Kaufmann K.C., Favero F.D.F., de Vasconcelos M.A.M., Godoy H.T., Sampaio K.A., Barbin D.F. (2019). Portable NIR spectrometer for prediction of palm oil acidity. J. Food Sci..

[48] Xiao M., Ma Y., Feng Z., Deng Z., Hou S., Shu L., Lu Z. (2018). Rice blast recognition based on principal component analysis and neural network. Comput. Electron. Agric..

[49] Dogantekin E., Dogantekin A., Avci D. (2010). An automatic diagnosis system based on thyroid gland: ADSTG. Expert Syst. Appl..

[50] Goel A., Srivastava S.K. Role of kernel parameters in performance evaluation of SVM. Proceedings of the 2016 Second International Conference on Computational Intelligence & Communication Technology (CICT).

[51] Nahato K.B., Nehemiah K.H., Kannan A. (2016). Hybrid approach using fuzzy sets and extreme learning machine for classifying clinical datasets. Inform. Med. Unlocked.

[52] Yang P., Wang D., Zhao W.B., Fu L.H., Du J.L., Su H. (2021). Ensemble of kernel extreme learning machine based random forest classifiers for automatic heartbeat classification. Biomed. Signal Process. Control.

[53] Cao Z., Wu X., Wu B., Zhang Z., Sun J. (2025). Combining UDT with XGBoost to identify the geographical origin of black beans by near-infrared spectroscopy. Curr. Res. Food Sci..

